# Clusterin Is Required for β-Amyloid Toxicity in Human iPSC-Derived Neurons

**DOI:** 10.3389/fnins.2018.00504

**Published:** 2018-07-25

**Authors:** Jacqueline P. Robbins, Leo Perfect, Elena M. Ribe, Marcello Maresca, Adrià Dangla-Valls, Evangeline M. Foster, Richard Killick, Paulina Nowosiad, Matthew J. Reid, Lucia Dutan Polit, Alejo J. Nevado, Daniel Ebner, Mohammad Bohlooly-Y, Noel Buckley, Menelas N. Pangalos, Jack Price, Simon Lovestone

**Affiliations:** ^1^Department of Psychiatry, University of Oxford, Oxford, United Kingdom; ^2^Department of Basic and Clinical Neuroscience, King’s College London, London, United Kingdom; ^3^Translational Genomics, Discovery Sciences, Innovative Medicines and Early Development Biotech Unit, AstraZeneca, Gothenburg, Sweden; ^4^Department of Old Age Psychiatry, King’s College London, London, United Kingdom; ^5^Nuffield Department of Medicine, Target Discovery Institute, University of Oxford, Oxford, United Kingdom; ^6^Innovative Medicines and Early Development Biotech Unit, AstraZeneca, Cambridge, United Kingdom

**Keywords:** Alzheimer’s disease, amyloid, neurodegeneration, clusterin, induced pluripotent stem cells, CRISPR/Cas

## Abstract

Our understanding of the molecular processes underlying Alzheimer’s disease (AD) is still limited, hindering the development of effective treatments, and highlighting the need for human-specific models. Advances in identifying components of the amyloid cascade are progressing, including the role of the protein clusterin in mediating β-amyloid (Aβ) toxicity. Mutations in the clusterin gene (CLU), a major genetic AD risk factor, are known to have important roles in Aβ processing. Here we investigate how CLU mediates Aβ-driven neurodegeneration in human induced pluripotent stem cell (iPSC)-derived neurons. We generated a novel CLU-knockout iPSC line by CRISPR/Cas9-mediated gene editing to investigate Aβ-mediated neurodegeneration in cortical neurons differentiated from wild type and CLU knockout iPSCs. We measured response to Aβ using an imaging assay and measured changes in gene expression using qPCR and RNA sequencing. In wild type neurons imaging indicated that neuronal processes degenerate following treatment with Aβ_25-35_ peptides and Aβ_1-42_ oligomers, in a dose dependent manner, and that intracellular levels of clusterin are increased following Aβ treatment. However, in CLU knockout neurons Aβ exposure did not affect neurite length, suggesting that clusterin is an important component of the amyloid cascade. Transcriptomic data were analyzed to elucidate the pathways responsible for the altered response to Aβ in neurons with the CLU deletion. Four of the five genes previously identified as downstream to Aβ and Dickkopf-1 (DKK1) proteins in an Aβ-driven neurotoxic pathway in rodent cells were also dysregulated in human neurons with the CLU deletion. AD and lysosome pathways were the most significantly dysregulated pathways in the CLU knockout neurons, and pathways relating to cytoskeletal processes were most dysregulated in Aβ treated neurons. The absence of neurodegeneration in the CLU knockout neurons in response to Aβ compared to the wild type neurons supports the role of clusterin in Aβ-mediated AD pathogenesis.

## Introduction

Alzheimer’s disease (AD) is characterized by the neuropathological features of amyloid plaques and neurofibrillary tangles (Glenner and Wong, 1984). The generation of excess intracellular β-amyloid (Aβ), or certain forms of oligomeric Aβ, is thought to be an early event in AD pathology. Aggregation of this protein initiates a complex cascade resulting in tau hyperphosphorylation, synaptic disruption, neuronal loss, and cognitive decline (Hardy and Selkoe, 2002). The neurotoxicity of Aβ has been extensively investigated in rodent studies, where it has been shown to induce increased activity of tau kinases such as Glycogen Synthase Kinase-3 (GSK-3), impair neuronal plasticity, and trigger neuronal cell death (Bloom, 2014; Spires-Jones and Hyman, 2014). However, these previous studies have limitations due to the use of rodent models in what is almost exclusively a human disease (Gunn-Moore et al., 2017). Indeed, transgenic rodent models prove remarkably resistant to Aβ, showing little tau phosphorylation or neuronal loss in the context of considerable excess Aβ generation and plaque pathology. Human *in vitro* models have great potential for studying human-specific mechanisms, and in a 3D cell culture system human induced pluripotent stem cell (iPSC)-derived neurons carrying familial AD mutations produced increased levels of Aβ and induced GSK-3 dependent tau phosphorylation and aggregation (Choi et al., 2014). This raises the prospect of improved *in vitro* models using human cells to determine the function of specific genes and processes involved in AD pathogenesis.

Clusterin (or apolipoprotein J) was first suggested to have a role in AD when it was found to be increased in the hippocampi of AD patients’ post-mortem (May et al., 1990). Subsequently, genome-wide association studies (GWAS) identified a genetic variant in the clusterin gene (CLU) associated with AD (Harold et al., 2009; Lambert et al., 2009), and proteomic studies found clusterin as a biomarker indicative of disease state in the blood (Thambisetty et al., 2010). The functional role of clusterin in AD, however, is not known, although a number of possible mechanisms for this highly pleiotropic protein have been suggested. Clusterin is an apolipoprotein, and along with apolipoprotein E (APOE) is involved in the transport of cholesterol, which has known effects on AD susceptibility (Yu and Tan, 2012). It also has an established role in the regulation of the complement pathway and may therefore have a role in mediating the neuroinflammatory component of AD (Kirszbaum et al., 1992). Clusterin might also be involved in the activation of microglia and recently has been shown to bind to the TREM2 receptor on microglia (Yeh et al., 2016).

Despite the role of CLU as a risk gene in AD being largely considered to be linked to inflammation or cholesterol metabolism (the Aβ-independent pathways in AD), as a glycoprotein with some chaperone properties it has been found to bind Aβ, and therefore one potential role of clusterin is in the clearance pathway of Aβ fibrils and peptides (Ghiso et al., 1993; Zlokovic et al., 1996). In rodent models, knockout of the CLU gene reduces the amount of fibrillar, but not total, Aβ in mice carrying the human APP (mutant) gene, and some transgene dependent phenotypes, such as dystrophic neurite number, are reduced in the absence of clusterin (DeMattos et al., 2002). In summary, clusterin has many biological functions and, not surprisingly therefore, has many potential roles in AD pathogenesis, including a possible role in Aβ induced neurotoxicity.

Previously a pathogenic pathway was identified that places clusterin as an important signal between oligomeric forms of Aβ peptide and phosphorylation of tau protein (Killick et al., 2014). Specifically, Aβ peptides increased intracellular clusterin in rodent neurons, and knockdown of CLU protected these cells from Aβ-induced cell death. Dickkopf-1 (DKK1) expression, a known negative regulator of Wnt signaling (Caricasole et al., 2004; Purro et al., 2014), was found to increase in response to Aβ, but not in CLU knock down neurons. In an attempt to further delineate this pathway, Aβ and DKK1 proteins were shown to each induce changes in gene expression that were also evident in animal models of amyloidopathy but not tauopathy, and in post-mortem brain in AD and Down’s syndrome but not other neurodegenerative disease (Killick et al., 2014). In the present study these observations are extended from rodent to human models, using iPSC-derived cortical neurons lacking CLU together with an isogenic control line to study neurodegenerative processes. Further evidence is provided for a role of clusterin in Aβ induced cell stress through development of a neurodegeneration assay, as well as investigating the effect of clusterin on cell function through transcriptomics and pathway analysis.

## Materials and Methods

### iPSC Culture

iPSCs were generated as previously described from human keratinocytes taken from hair samples of a healthy adult male (Cocks et al., 2014). Cells were reprogrammed from keratinocyte culture by exogenous expression of four reprogramming factors, *OCT3*/*4, SOX2, cMYC, KLF4*, using Sendai virus vector (SeVdp-iPS) (Nishimura et al., 2011). Cells were maintained in Essential 8 medium (Life Technologies) on Geltrex (Life Technologies) matrix as an adherent monolayer.

### Neuronal Differentiation

**FIGURE 1:**
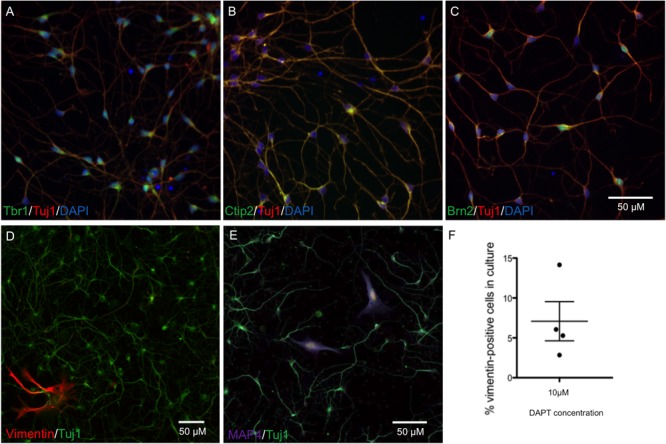
Characterization of neurons at day 28 by immunocytochemistry shows cortical markers Tbr1, Ctip2, and Brn2, and a low percentage of proliferating cells. Representative neuronal cultures **(A–C)** show Tbr1+, Ctip2+, Brn2+ neurons in green, Tuj1+ in red, and DAPI-stained nuclei in blue. Representative neuronal cultures **(D,E)** show Tuj1+ neurons in green and markers for Vimentin+ dividing cells in red or Map4+ dividing cells in purple. Neurons from *n* = 4 differentiations treated with DAPT are shown in **(F)**, with an average of 5% proliferative cells remaining in neuronal cultures. Scale bars represent 50 μm.

The protocol for neuronal differentiation was adapted from Shi et al. (2012). iPSCs were passaged at high density with Versene (Lonza), maintaining cells as clusters. After 24 h, SMAD inhibitors Dorsomorphin (1 μM) and SB431542 (10 μM) (Sigma) were added to each well in 50% N2 medium (N2 supplement in DMEM/F12 + 1% glutamax) and 50% B27 medium (B27 supplement in neurobasal medium + 1% glutamax) (all Life Technologies). N2/B27 medium and SMAD inhibitors were replaced every day for 7 days. Cells were dissociated with Accutase and replated in N2/B27 medium with 10 μM of Y-27632 ROCK inhibitors (ROCKi; Abcam) on days 8, 13, 16, and 19. Neural progenitors were passaged onto laminin (1 μg/cm^2^) for terminal differentiation of neurons at day 21 at 20,000 cells/cm^2^ in B27 medium + 10 μM DAPT + 10 μM ROCKi. After 48 h incubation media was replaced with B27. Cells were maintained in B27 medium for up to 45 days with a 50% media change every 3 days. Neurons generated from this protocol exhibited cortical lineage markers Brn2, Tbr1, and Ctip2 from day 28 (**Figures 1A–C**), and normal neuronal electrical activity from day 55 (Kathuria et al., 2017). The numbers of 4′,6-diamidino-2-phenylindole (DAPI)-stained nuclei in the cultures also staining positive for anti-vimentin, a marker for proliferative non-neuronal cells, were quantified, and found to make up 5% of cultures across an average of four differentiation experiments (**Figures 1D–F**). All antibodies are listed in Supplementary Table S1.

### Aβ Assay

In this project Aβ_25-35_ peptide was used in addition to Aβ_1-42_ oligomers, which due to its lower propensity to aggregate and its simpler preparation procedure offered reliable preparations of soluble Aβ. However, although this peptide has been reported as the cytotoxic fragment of the amyloid protein (Yankner et al., 1990), it is not typically produced by APP and so Aβ_1-42_ is the preferred length in terms of physiological relevance. Amyloid β-Protein (25–35) trifluoroacetate salt (Bachem) was solubilized in sterile water at 2 mg/ml and incubated for 2 h at 37°C. Amyloid β-Protein (35-25) trifluoroacetate salt (Bachem) was used as the control peptide for Aβ_25-35_ treatments. Aβ_1-42_ peptide was purchased from Dr. David Teplow (UCLA, Los Angeles, CA, United States) and was prepared as described in Tizon et al. (2010). Briefly, it was first resuspended in 100% 1,1,1,3,3,3 hexafluoro-2-propanol (HFIP) at a final concentration of 1 mM. The peptide was homogenized using a Teflon plugged Hamilton syringe and HFIP was evaporated in a SpeedVac. Aβ_1-42_ stocks were frozen at -20°C until 48 h before treatment. Aβ_1-42_ was resuspended in dimethylsulfoxide (DMSO) at 5 μM and sonicated for 10 min. Aβ_1-42_ oligomers were prepared by dilution in phosphate buffered saline (PBS) to 400 μM and 2% sodium dodecyl sulphate (SDS) in H_2_0 was added. After 24 h incubation at 37°C PBS was added to a final concentration of 100 μM and Aβ_1-42_ oligomers were incubated at 37°C for 18 h before adding to cell media. Ultracentrifugation and western blotting of 10 μM Aβ_1-42_ oligomeric preparations showed five different sized proteins present, with the predominant form of Aβ at 18.4 kDa (**Figure 3C**). DMSO was used as a control for Aβ_1-42_ treatment.

**FIGURE 2:**
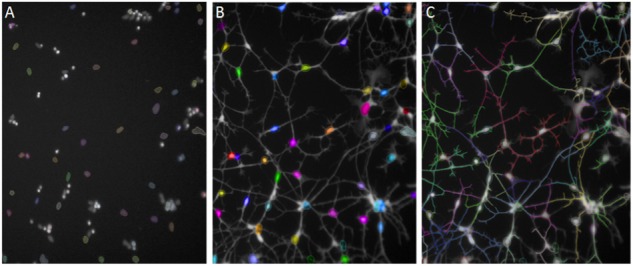
Automated Neuronal Profiling by imaging software at 20× magnification. **(A)** Nuclei are selected based on size and intensity in Hoechst channel. **(B)** Nuclei selected in Step A are located in the Calcein AM channel as cell bodies and selected in color by the software. **(C)** Neuronal processes extending from the cell bodies with Calcein AM stain are traced and the total length of the neurites per cell are measured in numbers of pixels.

After 48 h of Aβ exposure cells were stained with Calcein Green AM (CAM; Life Technologies) live cell dye at 1 μg/ml and membrane permeable Hoechst at 0.01 μg/ml and incubated at 37°C for 20 min. Thermo Scientific CellInsight Personal Image Cytometer with iDEV software or PerkinElmer Operetta High Content Imager with Harmony software was used to develop imaging assays. The neurite length assay was originally developed from the Neuronal Profiling BioApplication in iDEV. Images were taken at 10× magnification. Intact, Hoechst-stained nuclei were selected in channel 1 (350 nm; **Figure 2A**) and cell bodies stained with CAM were identified in channel 2 (488 nm; **Figure 2B**). Neurites protruding from cell bodies were then traced by the software (**Figure 2C**) and the average length across the well measured. Differences in neurite length between treatment conditions and cell lines were assessed using unpaired, two-tailed *t*-tests with 95% confidence intervals.

### Western Blotting

Cells were loaded 1:1 with 4× Laemmli Sample buffer for separation on NuPAGE^®^ Novex^®^ 4–12% Bis-Tris gels (Life Technologies). Anti-clusterin (1:1000; Santa Cruz) and anti-α-tubulin (1:10000; Abcam) were detected with fluorophore conjugates AlexaFluor680 or AlexaFluor800 (1:5000; Life Technologies) using an infrared scanner (LI-COR Biosystems, Cambridge, United Kingdom). The integrated density for clusterin bands from four independent experiments was normalized against α-tubulin to account for varying protein loads and unpaired, two-tailed *t*-tests were performed to compare Aβ and control. All antibodies for western blotting are included in Supplementary Table S1.

### Real-Time Polymerase Chain Reaction (qRT-PCR)

RNA extraction was performed from cells collected in TRIzol solution (Life Technologies), and RNA was isolated by the TRIzol method and clean up of RNA was performed using the RNeasy kit (Qiagen) according to manufacturers’ instructions. Complimentary DNA (cDNA) was synthesized using SuperScript^®^ III Reverse Transcriptase (Life Technologies). qPCR reactions were carried out on a Chromo4^TM^ Real-Time PCR detector (Bio-Rad) using SYBR^®^ Green PCR Master Mix (Life Technologies). Each reaction was carried out with a technical replicate and with three biological replicates for each target gene and three housekeeping genes. The Pfaffl mathematical model for relative transcript quantification was used for data analysis using cycle threshold (CT) values and efficiency values (Pfaffl, 2001). Comparisons of relative expression using the Pfaffl method in Aβ-treated cells compared to controls were performed using unpaired, two-tailed *t*-tests with 95% confidence intervals.

### CRISPR/Cas9 Genome Editing of iPSCs

For knockout of the CLU gene exon 3 was selected due to its presence in all isoforms of the gene in human cells. A specific gRNA sequence was selected by bioinformatics analysis where a full list of potential gRNA sequences was ranked based on low off-target cleavage potential, number of bases to a PAM site, and predicted efficiency. Homologous recombination after CRISPR/Cas9 cleavage was performed to insert a targeting construct including a 2A GFP and a floxed neomycin selection cassette (all sequences included in Supplementary Table S2). Transfection of iPSCs with cas9-GFP constructs was performed using Lipofectamine LTX (Life Technologies). Clonal iPSC populations that had been selected by 150 μg/ml neomycin were assessed for the integration of the CLU targeting construct by PCR using primers both in the exon sequence and in the targeting construct. The presence of the insertion of a cassette on one CLU allele and an out of frame deletion in the other CLU allele, and the lack of any off-target integration of the cassette, was confirmed by Targeted Locus Amplification (TLA) genotyping (see Supplementary Figures S1, S2) performed by Cergentis, The Netherlands (de Vree et al., 2014). Decreased expression of CLU mRNA was confirmed by the design of qPCR primers binding at exon 2 and exon 3 of the CLU gene, the region identified through sequencing to be absent in both alleles of the CLU knockout line. The iPSC line was subject to quality control experiments to confirm pluripotent potential. iPSCs were fixed for immunocytochemistry and stained with antibodies for pluripotency: Nanog (Abcam), Oct3/4 (SantaCruz Biotech), TRA-1-81 (Abcam), and SSEA4 (Abcam), and differentiated into the three germ layers: ectoderm (βIII tubulin), mesoderm (smooth muscle actin), and endoderm (α-fetoprotein). Antibodies listed in Supplementary Table S1.

### RNA Sequencing

Aβ_1-42_ was added to wild type and CLU-/- neurons in six well plates for 48 h at concentration 1 μM. Lysates were collected from Aβ-treated cells and untreated controls, with three biological replicates per group, in TRIzol for RNA extraction. Total RNA was isolated using RNeasy Kit (Qiagen), Library preparation, and RNAseq was carried out by Wellcome Trust Centre for Human Genetics, University of Oxford. RNA samples were first analyzed on a tape-station for quality control. TruSeq RNA Library Preparation Kit (Illumina) was used and mRNA was enriched by polyA selection. RNAseq was carried out using on the Illumina HiSeq 4000 with 10 samples per lane, approximately 24 million 75 bp paired-end reads per sample.

Preprocessing was performed using the software tool Trimmomatic to quality trim the reads and remove adapters (Bolger et al., 2014 ^1^). STAR was used with default settings to align the reads (Dobin et al., 2013) to the human genome references assembly (build GRCH38.p5). Differential gene expression analysis was then performed using DESeq2 (Love et al., 2014). All fold changes were included in the analysis if the change in expression was significant once corrected for multiple comparisons. The gene ontology (GO) analyzer GOseq (Young et al., 2010) was used to detect the over/under representation of GO terms and Kyoto Encyclopedia of Genes and Genomes (KEGG) pathways in the differentially expressed gene sets. Overlapping GO terms were removed using REViGO (Supek et al., 2011).

## Results

### Aβ Reduces the Length of Neuronal Processes and Increases Intracellular Clusterin Levels in Human Neurons

**FIGURE 3:**
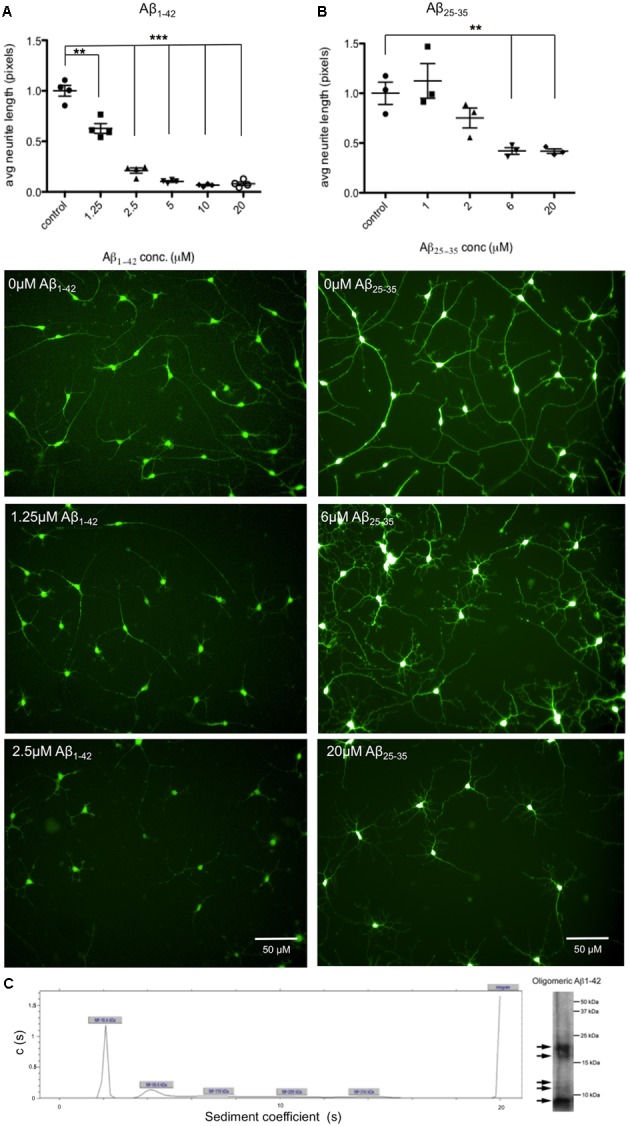
Aβ treatment decreases length of neuronal processes. Neurons from *n* = 3 differentiation experiments were treated with either Aβ_25-35_ peptides or Aβ_1-42_ oligomers at a range of concentrations (0–20 μM) and stained with the live cell dye calcein acetoxymethyl (Calcein AM). Neurite length was measured by automated imaging software and unpaired *t*-tests compared neurite length in treated cells to untreated controls. **(A)** Representative images of neurons treated with Aβ_1-42_ oligomers at 0 μM, 1.25 μM, and 2.5 μM for 48 h. **(B)** Representative images of neurons treated with Aβ_25-35_ peptides at 0 μM, 6 μM, and 20 μM for 48 h. (^∗∗^*p* < 0.005, ^∗∗∗^*p* < 0.001). Scale bars represent 50 μm. **(C)** Characterization of Aβ_1-42_ oligomers with analytical ultracentrifugation. Ultracentrifugation and western blotting shows sizes of proteins present in Aβ_1-42_ sample preparations.

Aβ induced phenotypes prior to neuronal cell death were investigated through the development of an automated assay of neurite length. As in previous studies (Killick et al., 2014), cells were exposed to both Aβ_25-35_ peptides and full length Aβ_1-42_ oligomers for 48 h. Neurons were differentiated for 35 days prior to treatment and then stained with Calcein AM to identify live cells together with the nuclear stain Hoechst and the length of neuronal processes measured using automated cell imaging. In order to determine the optimal concentration of peptide and duration of exposure, seeking relatively low, near physiological, levels of Aβ exposure, cells were treated with Aβ concentrations up to 20 μM. A concentration of 1.25 μM Aβ_1-42_ treatment for 48 h was sufficient to cause a significant reduction in neurite length (**Figure 3A**), or 6 μM of Aβ_25-35_ treatment (**Figure 3B**). This established a minimum concentration dose to trigger a degeneration response from iPSC-derived neurons. Since 20 μM Aβ_25-35_ treatment caused observable cell toxicity a cellular viability assay was performed to investigate this, and increased apoptosis as measured by cleaved caspase 3 staining, was detected at 10 μM Aβ_25-35_ for 24 h (data included in Supplementary Figure S3).

**FIGURE 4:**
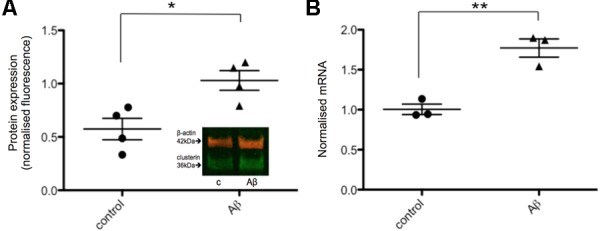
Intracellular clusterin levels are higher in Aβ-treated neurons. **(A)** Neurons from four wells from four independent differentiations were treated with 20 μM Aβ_25-35_ for 48 h and intracellular protein levels were measured by western blotting and normalized to α-tubulin levels. **(B)** Neurons from three independent differentiations were treated with 20 μM Aβ_25-35_ for 48 h, consistent with the treatment by Killick et al. (2014), and gene expression levels were measured by qPCR. Expression of genes was quantified relative to housekeeper genes. (^∗^*p* < 0.05, ^∗∗^*p* < 0.005).

Next, the effects of Aβ on clusterin in human neurons was investigated in order to compare to rodent neurons derived from fetal brain as previously reported (Killick et al., 2014). Neurons derived from iPSCs were treated with 20 μM Aβ_25-35_ for 48 h after which both intracellular clusterin protein levels and expression of genes previously shown to be responsive to Aβ were assessed. Neurons exposed to Aβ_25-35_ demonstrated increased levels of intracellular clusterin compared to untreated neurons (**Figure 4A**), as reported previously in fetal rat neurons. CLU gene expression was measured using qPCR with expression levels normalized to three housekeeping genes and found CLU expression significantly increased after 48 h of Aβ exposure (**Figure 4B**).

**FIGURE 5:**
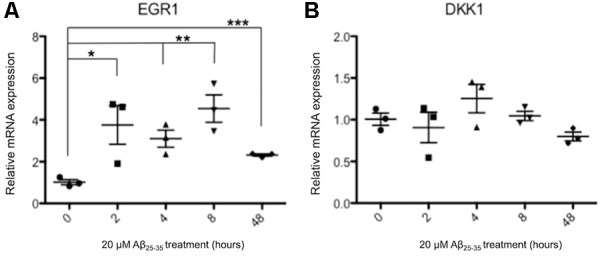
Changes in EGR1 **(A)** and DKK1 **(B)** gene expression in Aβ-treated neurons. Neurons from three independent differentiations were treated with 20 μM Aβ_25-35_ peptides for up to 48 h and expression of genes quantified by qPCR relative to housekeeper genes. The relative gene expression levels are normalized to the pre-exposure baseline ± SEM, and unpaired *t*-tests were performed for all four time points. (^∗^*p* < 0.05, ^∗∗^*p* < 0.005, ^∗∗∗^*p* < 0.001).

Previously Aβ has been reported to induce the expression of DKK1 (Caricasole et al., 2004; Purro et al., 2012, 2014), and both Aβ and DKK1 were found to independently induce EGR1, and knockdown of EGR1 prevented Aβ induced toxicity (Killick et al., 2014). Therefore, it was investigated whether the same gene expression changes were also induced in human neurons treated with 20 μM Aβ_25-35_ for up to 48 h. EGR1 expression was significantly increased from 2 h of Aβ exposure, which was continued through to 48 h (**Figure 5A**). The highest increase in EGR1 expression was a 4.5-fold change after 8 h of Aβ compared to control neurons. However, DKK1 expression was not affected by Aβ-treatment at the time points investigated in human neuronal cultures (**Figure 5B**).

### Generation of a CLU Knockout Cell Line

**FIGURE 6:**
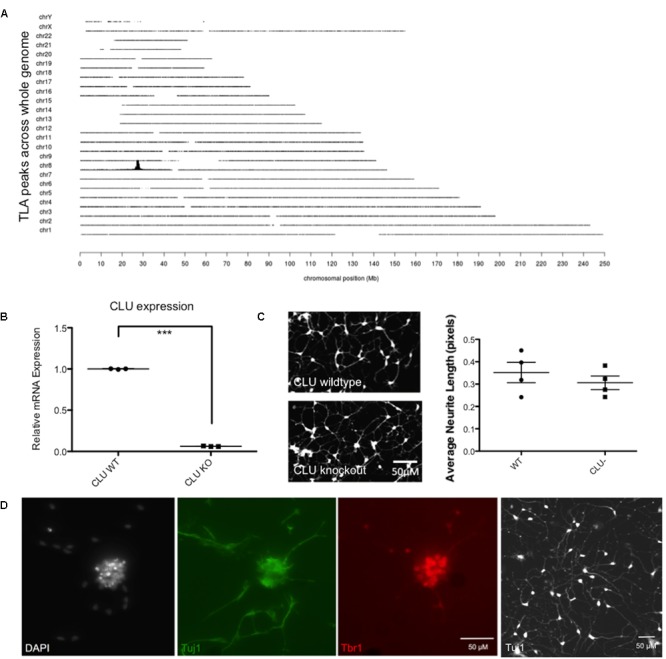
Validation of a CLU knockout (KO) iPSC line. **(A)** Whole genome coverage plot shows a single identified integration site in the CLU gene on Chromosome 8 and no off target integrations elsewhere in the genome. Targeted Locus Amplification (TLA) technology by Cergentis with the transgene specific primer pair shows chromosomal positioning of targeting construct integration. **(B)** Neurons differentiated from CLU knockout iPSCs expressed significantly reduced CLU mRNA (*p* < 0.0001). qPCR of CLU relative to housekeeping gene in wild type compared by *t*-test to knockout neurons for three technical replicates from one differentiation experiment. **(C)** Wild type neurons and CLU knockout neurons differentiated for 35 days and imaged using Calcein AM live cell stain. Neurite length assay shows the average length of neuronal processes from three independent differentiations of CLU knockout neurons compared to wild type neurons. **(D)** Representative neuronal cultures show DAPI-stained nuclei in white, Tuj1+ neurons in green, and cortical marker Tbr in red. A lower magnification view of Tuj1+ CLU knockout neurons is also shown. Scale bars represent 50 μm.

Having shown that human neurons derived from iPSCs are responsive to Aβ and, like rodent neurons, generate an increase in intracellular clusterin and an increase in expression of EGR1, a human cell line with the CLU gene deleted was developed using CRISPR/Cas9 to specifically disrupt CLU expression. A targeting construct containing a 2A GFP floxed PGK neo cassette was integrated by CRISPR/Cas9 mediated homologous recombination into exon 3 of the CLU gene. Successful targeting of one allele and an out of frame mutation of the other allele was confirmed by TLA, and off-target integration of the targeting cassette was also excluded (**Figure 6A**), confirming that the CLU gene had been knocked out of chromosome 8 only in the iPSC line. CLU mRNA expression was shown by qPCR to be reduced by 94% in the CLU knockout cells compared to wild type (*p* = 0.0018; **Figure 6B**). Clusterin knockout iPSCs differentiated normally into post-mitotic neurons, showing analogous differentiation rate and morphology to isogenic wild type neurons (**Figure 6C**). Immunocytochemistry for neuronal Tuj1 and cortical marker Tbr1 demonstrated cortical lineage of the neurons upon differentiation (**Figure 6D**).

### CLU Knockout Neurons Are Not Vulnerable to Aβ Insult

**FIGURE 7:**
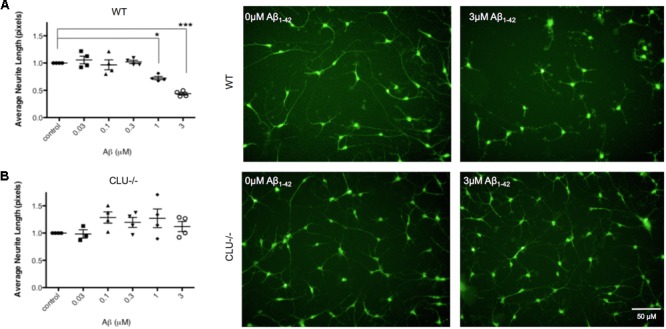
Neurite length is decreased in response to Aβ_1-42_ in wild type (WT) neurons but not in CLU knockout (CLU-/-) neurons. Neurons from three independent differentiations of both WT and CLU-/- lines were treated with 0–3 μM Aβ_1-42_ oligomers for 48 h neuronal processes and neurite length was measured by automated image analysis. **(A)** Neurite length in WT neurons treated with 0–3 μM Aβ_1-42_ and representative images at 0 and 3 μM. **(B)** Neurite length in CLU-/- neurons treated with 0–3 μM Aβ_1-42_ and representative images at 0 and 3 μM. (^∗^*p* < 0.05, ^∗∗^*p* < 0.005). Scale bars represent 50 μm.

Neurons derived from CLU knockout iPSCs and their isogenic control iPSC line were studied to examine the role of clusterin in Aβ-mediated neurodegeneration. Wild type and CLU knockout neurons were differentiated for 35 days and treated with Aβ_1-42_ oligomeric preparations for 48 h. There was no difference in neurite length between untreated wild type and CLU knockout neurons (**Figure 6B**). Wild type neurons were responsive to Aβ, with a significant decrease in neurite length induced by 1 μM Aβ_1-42_ exposure (**Figure 7A**) as in previous experiments. In contrast, CLU knockout neurons showed no significant response to Aβ_1-42_ treatment up to 3 μM (**Figure 7B**). At higher concentrations (3 μM for 48 h), Aβ_1-42_ resulted in over 50% decrease in neurite length in wild type neurons (*p* = 0.0005), but still showed no effect on neurite length in CLU knockout cells.

### Clusterin Deletion Significantly Alters Expression of Previously Identified Aβ Responsive Genes

Clusterin is a highly pleiotropic protein and understanding of the proteins interactions with the Aβ protein are still evolving. Since neurons lacking the clusterin protein showed protection against neurodegeneration, a transcriptome wide expression study was conducted to map the effects of clusterin on pathways active in human neurons.

Previously, to identify genes associated with Aβ-DKK1-driven neurotoxicity, rodent neurons were treated with either DKK1 or Aβ and the data analyzed to identify gene dysregulation common to both exposures. Whilst many genes were common to both exposures, the most striking observation was that of the top 10 most dysregulated genes from each exposure, five (EGR1, CCND1, KLF10, FOS, and NAB2) were common to both DKK1 and Aβ (Killick et al., 2014). Therefore, whether these genes were dysregulated in the human neurons was tested in the CLU knockout compared to WT neurons. Four of these five genes were significantly dysregulated in the CLU knockout neurons: CCND1 (*p* < 0.001), KLF10 (*p* < 0.001), FOS (*p* < 0.001) and EGR1 (*p* < 0.05) (**Table 1**).

**Table 1:** Differential expression in human CLU knockout neurons compared to wild type of the top five genes dysregulated by Aβ and DKK1 in rodent cells.

Symbol	Ensembl code	Fold change	*p*-value	p-adj
CCND1	ENSG00000110092	3.00192	1.89E-92	2.97E-89
KLF10	ENSG00000155090	3.23701	7.19E-29	5.71E-27
FOS	ENSG00000170345	1.91373	0.000277	0.00147
EGR1	ENSG00000120738	0.60533	0.033569	0.086988
NAB2	ENSG00000166886	0.78814	0.167693	0.302385

*The five genes are shown with the fold change, *p*-value, and the adjusted *p*-value taking into account the number of genes studied in the analysis between the CLU knockout line and the WT line.*

### Differentially Expressed Pathways in CLU Knockout Neurons Reveal Major Transcriptomic Differences

**FIGURE 8:**
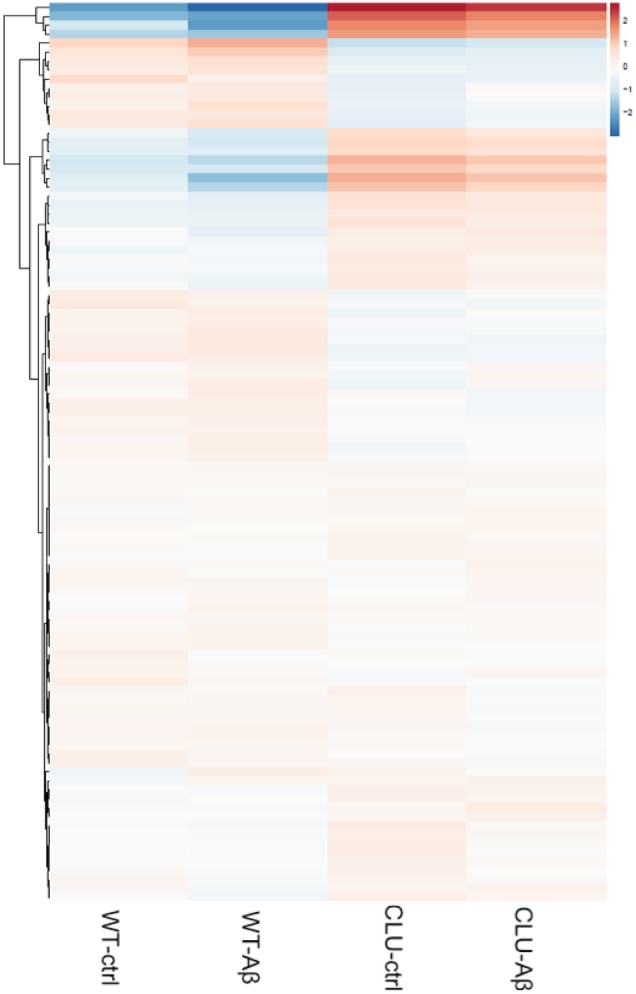
A heatmap of regularized log transformed counts of all genes in the four conditions. A plot of the regularized log transformed counts for all genes measured shows a greater difference in gene expression in CLU-/- compared to wild type neurons, than between treatment groups across the whole genome. Red indicates upregulation; blue indicates downregulation of gene set.

In order to further explore the pathways responsive to clusterin, a differential transcript study of genes was performed in CLU knockout neurons (control, untreated, and 1 μM Aβ_1-42_ treated) compared to clusterin wild type neurons (control, untreated, and 1 μM Aβ_1-42_ treated) using RNAseq. Analysis of all genes revealed 9664 genes differentially regulated between the WT line and the CLU knockout line after 35 days of neuronal differentiation (**Figure 8**). The gene set heatmap also showed an observable difference in gene expression between the treatment groups for both the WT and CLU knockout neurons, albeit less striking than that consequent to loss of clusterin.

**FIGURE 9:**
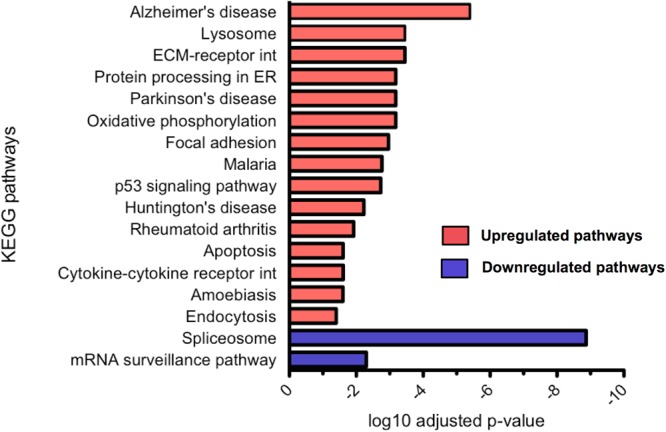
Significantly up and downregulated KEGG pathways in CLU knockout neurons compared to WT neurons. KEGG pathway enrichment analysis of upregulated genes and downregulated genes, with *p*-value corrected for multiple analyses. 15 pathways were significantly upregulated and 2 significantly downregulated. (ECM, extracellular matrix; int, interaction; ER, endoplasmic reticulum)

Differential gene expression analysis was carried out using DESeq2 and differentially expressed genes were tested for KEGG pathway enrichment by GOseq. Significantly upregulated and downregulated pathways in CLU knockout untreated neurons compared to WT untreated neurons were ranked by significance (**Figure 9**). The most dysregulated KEGG pathway in CLU knockout neurons compared to CLU wild type was the AD pathway (*p* = 0.000004; Supplementary Figure S4), with other gene sets linked to neurodegenerative diseases also significantly dysregulated. However, in the CLU knockout neurons treated with Aβ the AD pathway is downregulated compared to the CLU knockout untreated (*p* = 5.84E-07), along with other neurodegenerative disease pathways. Therefore, the genes involved in this disease pathway could be mediating the neuroprotective effect observed in Aβ-treated CLU knockout neurons. The dysregulation of other neurodegenerative disease pathways suggests a role for CLU in neurodegeneration processes beyond its direct interaction with Aβ.

### Molecular Functions Related to the Cytoskeleton May Be Predominantly Responsible for Aβ-toxicity in iPSC-Derived Neurons

**FIGURE 10:**
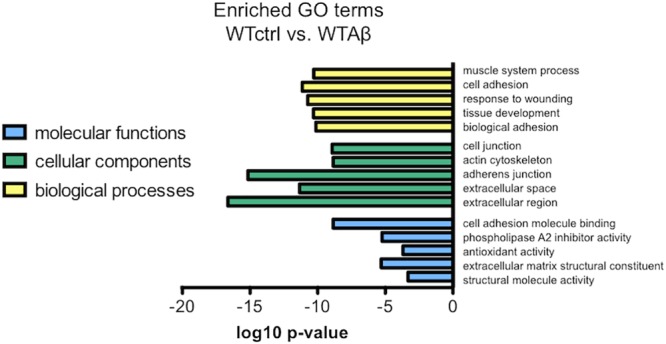
The most enriched GO terms for Aβ-treated WT neurons compared to control neurons. The top 5 downregulated molecular functions, cellular components, and biological processes in negatively regulated genes from DESeq2 analysis in Aβ-treated neurons compared to control. There were no significantly upregulated gene sets.

To investigate the molecular functions underlying the effect of Aβ in wild type human neurons, a function that was abrogated in the CLU knockout neurons, gene expression in Aβ-treated neurons was compared to that in untreated neurons. Differentially regulated genes were assessed for GO enrichment by GOseq, extracting both up and down regulated pathways in the categories: biological processes, cellular components, and molecular functions. The top 5 significantly downregulated GO terms in Aβ-treated wild type neurons (WTAβ) compared to untreated neurons (WTctrl) are ranked in **Figure 10**. There were no significantly upregulated GO pathways. When this analysis was repeated in neurons lacking CLU none of the same biological processes were significantly altered in the CLU knockout neurons when treated with Aβ, and structural molecule activity was the only overlapping molecular function. This suggests that these biological processes and molecular functions may contribute to the Aβ-induced, clusterin-mediated neurodegeneration process. The major differentially regulated pathways are largely dominated by cellular structural functions, including cell adhesion, actin cytoskeleton, and extracellular structure.

## Discussion

The misprocessing and accumulation of Aβ is considered one of the earliest events in AD pathogenesis. Multiple lines of evidence suggest that it is the soluble and probably intracellular oligomeric forms of Aβ that induce synaptotoxicity in rodent neurons, rather than the aggregated fibrillar forms of Aβ found in amyloid plaques (Shankar et al., 2007, 2008). Similar findings have been reported in iPSC-derived human neurons treated with Aβ derived from Chinese hamster ovary cells stably expressing mutant human APP, inducing synaptotoxicity (Nieweg et al., 2015). Protecting against soluble Aβ peptide-induced neurotoxicity could be a useful therapeutic strategy for AD, and therefore elucidating the mechanisms that drive this Aβ-mediated degeneration is important. The present study confirms that human iPSC-derived neurons respond to both Aβ_1-42_ oligomers and Aβ_25-35_ peptides with signs of neurodegeneration after only 48 h of exposure. The 1.25 μM for Aβ_1-42_ and corresponding toxicity of 5 μM of Aβ_25-35_ established in the neurite length assay (see **Figure 3**) can be considered in the physiologically relevant range, with Näslund et al. (2000) estimating levels of Aβ_1-40_ and Aβ_1-42_ in the cortex of post-mortem AD brains at 1–3 μM. Toxic soluble Aβ aggregates have been found to be at highest concentration at the plaques, causing a gradient of toxicity to synapses out from the plaque core (Brody et al., 2017). Our *in vitro* assay with synthetic Aβ additions is a useful tool for modeling neuronal response to Aβ in brain regions with high amyloid load and the area surrounding amyloid plaques. Furthermore, the human neurons demonstrated the same response to Aβ as previously shown in rodent neurons, with Aβ treatment increasing intracellular clusterin levels (Killick et al., 2014), although, in this study Aβ also induced CLU gene expression.

In order to further explore the role of clusterin in AD, a novel CLU knockout iPSC line was generated using CRISPR/Cas9 precise genome editing, and successfully differentiated into cortical neurons allowing comparison to isogenic cells possessing an unedited CLU gene. Using these cells, a protective role for clusterin was found in a human neuronal model of the amyloid cascade, which supports the importance of clusterin in amyloid-dependent AD pathogenesis previously established in post-mortem tissue and animal models (May et al., 1990; DeMattos et al., 2002; Thambisetty et al., 2010; Narayan et al., 2011). Human neurons lacking the CLU gene do not demonstrate observable neurodegeneration as measured by neurite length when exposed to Aβ oligomers at concentrations that cause significant neurite reduction in wild type neurons.

Just as in previous rodent neuronal studies (Killick et al., 2014), this study shows that in human neurons *in vitro* clusterin accumulates intracellularly in response to Aβ exposure. However, in addition to these previous studies, this study found not only an increase in cellular distribution of clusterin but also an increase in gene expression. Interestingly, a lack of common mutations in the CLU gene associated with gene expression have led to suggestions that CLU may exert its effect through altering gene expression in response to insult (Guerreiro et al., 2010), or that at least some mutations associated with disease cause changes in clusterin distribution through trafficking (Bettens et al., 2015). These experimental results, first in rodent and now in human neurons, suggest both mechanisms – an insult induced change in expression and an insult induced change in distribution – may play roles in the clusterin mediated neurodegeneration in AD. Further studies investigating clusterin localization and how this affects neuronal survival under Aβ exposure conditions are needed to determine the role of clusterin in AD, and decipher why CLU SNPs contribute to AD risk.

Knockout of CLU expression protected human neurons from Aβ-induced phenotypes including neurite length, offering further evidence that clusterin is critically important in transducing Aβ induced neurotoxicity. The overlap observed between the Aβ neurotoxicity pathway genes established in rodent cultures (Killick et al., 2014) and the human CLU knockout neurons further supports the involvement of clusterin in Aβ-dependent neurodegeneration, and adds to the evidence suggesting this molecular cascade as a mechanism. Knockdown of EGR1 was previously found to prevent Aβ-induced toxicity and EGR1 has been previously reported to dysregulate key AD genes, including BACE and presenilin-2 (Renbaum et al., 2003; Killick et al., 2014; Qin et al., 2016). Further investigation of these transcription factors that are dysregulated by Aβ and CLU in a human iPSC-derived model of AD may help to elucidate their role in Aβ-dependent neurodegeneration.

The RNAseq data here provides a comprehensive overview of the transcriptome of differentiated CLU knockout and CLU wild type neurons, which exhibit contrasting responses to Aβ, and identifies several potential molecular functions and pathways that may be responsible for this phenotype. Interestingly, the gene expression profile of neurons lacking CLU proposes multiple pathways relating to neurodegeneration, suggesting that CLU may have a role in degeneration of neurons beyond a direct role in Aβ-mediated toxicity, and may be important for neuronal response to insult in multiple diseases. Neurons treated with Aβ had downregulation of pathways predominantly relating to the cytoskeleton, which may implicate these genes in the propagation of the Aβ cascade, although this finding may also be a consequence and not cause of this cascade.

Although iPSC derived neuronal cultures have many advantages over neuronal cultures derived from rodents, especially in models of disease where the animal seems resolutely resistant to the disease under investigation, there are limitations to the utility of these cells. For example, although Aβ-toxicity in human neuronal cultures is demonstrated, this may not reflect the Aβ-driven pathways measured in *in vivo* studies due to the simplicity of an almost purely neuronal model. *In situ* in the brain, clusterin is primarily expressed by astrocytes, and it is possible that the molecular mechanisms of Aβ induced neurodegeneration may be dependent on the presence of other cell types – most obviously neural cells of the inflammatory system. Indeed clusterin binds to the TREM2 receptor on microglia, an activity that has been suggested to facilitate uptake and turnover of Aβ (Yeh et al., 2017). One discrepancy between this study and previous observations and those of others, is the failure to observe an increase in DKK1 expression in human neurons in response to Aβ, and it may be that multiple cell types are necessary for the induction of this secreted wnt inhibitory factor. Therefore although differentiated neurons are useful to examine neuron-autonomous phenotypes as demonstrated by this study and others, it might be that complex cell models or models using other neuronal cell types will become more informative when modeling complex aspects of disease mechanisms (Choi et al., 2014; Haenseler et al., 2017).

The immature phenotype of these neurons may also be a limitation to studying Aβ-induced neurodegeneration in this model. Studies indicate that during reprogramming to an embryo-like iPSC state the original age of the somatic cells is lost, and ageing hallmarks are reset, including mitochondrial function and oxidative stress levels (Meissner et al., 2008; Lapasset et al., 2011; Rohani et al., 2014). Mature iPSC-derived neurons exhibit a similar transcriptomic profile to fetal neurons, but distinct from adult neurons. (Patani et al., 2012; Handel et al., 2016). Neurons matured for 150 days were found to express tau isoform profiles similar to brain samples from post-mortem patients (Iovino et al., 2015). This suggests that neurons differentiated for 35 days, as used here, may be suitable for studies utilizing exogenous Aβ but would be too immature to show certain key AD-related cellular responses.

More studies using human cells are now needed to establish the Aβ-dependent role of clusterin in AD. Determining the patterns of isoform expression and protein trafficking in response to Aβ exposure will be important, and may offer new insights into how CLU mutations identified from GWAS contribute to disease risk. Differentiating iPSCs into multiple cell types could produce a more accurate model of the cortical environment. Clusterin and APOE are predominantly synthesized and secreted by astrocytes in the brain (Xu et al., 2006), and therefore mixed culture models could transform our understanding of the roles of these apolipoproteins in AD.

## Conclusion

The development of a novel CLU knockout iPSC line is reported here, which has been successfully differentiated into cortical neurons. Neurons lacking CLU did not show neurodegeneration in response to Aβ, unlike CLU wild type neurons, placing CLU as a necessary effector of Aβ toxicity. This adds support to the protein clusterin having a contributing role in AD pathogenesis through its Aβ-dependent pathogenic action, and provides the first confirmation of this Aβ-initiated pathway in human neuronal cultures.

## Data Availability

The datasets used during the current study, not already available in the manuscript or supplementary material, are available from the corresponding author on reasonable request. The RNAseq dataset including the DESeq output and raw files is available on www.synapse.org, Project syn12216049.

## Author Contributions

JR was involved in study design, performed the experiments, and drafted the figures and the manuscript. LP performed RNAseq analysis and interpretation, and assisted with RNA extractions and qPCR. MM designed genome-editing strategy and experiment, and assisted with molecular biology. RK and ER were involved in study design, Aβ preparations, and assay development. PN assisted with neuronal differentiations and iPSC maintenance. MR conducted the quality control for the knockout iPSC line. LDP, AD-V, and EF assisted with RNA extractions and qPCR. AN helped with RNAseq data interpretation. DE was involved in RNAseq experiment design and imaging assay development. MB-Y was involved in genome-editing experiment design and support. NB helped with RNAseq data interpretation. MP was involved in the initial design of the study and data interpretation. JP conceived design of experiments, provided the cell line, and assisted with drafting the manuscript. SL conceived design of study, and was involved in data interpretation and critical revision of the manuscript. All authors read and approved the final manuscript.

## Conflict of Interest Statement

MM, MB-Y, and MP are shareholders and employees of AstraZeneca. The remaining authors declare that the research was conducted in the absence of any commercial or financial relationships that could be construed as a potential conflict of interest.

## Footnotes

1 usadellab.org
