# Supplementary material for: Clusterin Is Required for β-Amyloid Toxicity in Human iPSC-Derived Neurons
Source: Front Neurosci. 2018 Jul 25;12:504. doi: 10.3389/fnins.2018.00504 (PMC6068261; doi:10.3389/fnins.2018.00504)
Supplement: Supplementary file 1 [file Data_Sheet_1.docx]

Supplementary Material

Article Title

Jacqueline P. Robbins, Leo Perfect, Elena M. Ribe, Marcello Maresca, Adrià Dangla-Valls, Evangeline M. Foster, Richard Killick, Paulina Nowosiad, Matthew J. Reid, Lucia Dutan Polit, Alejo J. Nevado, Daniel Ebner, Mohammad Bohlooly-Y, Noel Buckley, Menelas N. Pangalos, Jack Price, Simon Lovestone^*^

*** Correspondence:** Simon Lovestone, simon.lovestone@psych.ox.ac.uk

# Supplementary Table 1

| Primary Antibody | Species, Dilution | Company |
| --- | --- | --- |
| α-fetoprotein | Rabbit, 1:500 | Abcam ab133617 |
| α-smooth muscle actin | Rabbit, 1:1000 | AbCam ab5694 |
| α-tubulin | Mouse,1:10000 | AbCam ab24610 |
| β-actin | Mouse, 1:10000 | AbCam ab8227 |
| BRN2 | Rabbit, 1:500 | SantaCruz sc28594 |
| Cleaved caspase-3 | Rabbit, 1:500 | Cell Signalling Technology 9664 |
| Clusterin | Mouse, 1:1000 | SantaCruz sc-5289 |
| CTIP2 | Rat, 1:500 | AbCam ab18465 |
| Map4 | Rabbit, 1:400 | GeneTex GTX110656 |
| Nanog | Rabbit, 1:1000 | Abcam ab109884 |
| Oct4 | Rabbit, 1:1000 | AbCam ab109884 |
| SSEA4 | Mouse, 1:500 | AbCam ab109884 |
| TBR1 | Rabbit, 1:500 | AbCam ab31940 |
| TRA-1-81 | Mouse, 1:500 | AbCam ab109884 |
| Tuj1 (Neuronal specific Biii tubulin) | Mouse, 1:1000 | Covance MMS-435P |
| Vimentin | Rabbit, 1:400 | Cell Signalling D21H3 |

| Secondary Antibody | Dilution | Company |
| --- | --- | --- |
| Alexa Fluor 680 donkey anti-rat | 1:10000/1:50000 | ThermoFisher A21096 |
| Alexa Fluor 800 donkey anti-mouse | 1:10000/1:50000 | ThermoFisher A32730 |
| Alexa Fluor 680 donkey anti-rabbit | 1:10000/1:50000 | ThermoFisher A10043 |
| Alexa Fluor 488 donkey anti-rabbit | 1:5000 | ThermoFisher A21206 |
| Alexa Fluor 488 donkey anti-mouse | 1:5000 | ThermoFisher A21202 |
| Alexa Fluor 594 donkey anti-rabbit | 1:5000 | ThermoFisher R37119 |
| Alexa Fluor 594 donkey anti-mouse | 1:5000 | ThermoFisher R37115 |

## Supplementary Table 2

| **guide RNAs** |  |
| --- | --- |
| **Name** | **Sequence** |
| CLU crispr1 F | accgacgtacttacttccctgat |
| CLU crispr1 R | aaacatcagggaagtaagtacgt |
| CLU crispr2 F | accgaaattcaaaatgctgtcaa |
| CLU crispr2 R | aaacttgacagcattttgaattt |
|  |  |
| **primers for targeting construct integration:** |  |
| **Name** | **Sequence** |
| CLU exon 3 | atcaggccccagtgttacag |
| CLU targeting construct | gtcctccttgaagtcgatgc |
|  |  |
| **Targeting plasmids:** |  |
| **Name** | **Sequence** |
| CLU-T2A-GFP | TTTCCAGTTATGATATGGTGTTATTAACTATAAGCACCACCTGTATGTTAGACCTCCAGAACATACTCCTCCTACCTGATGAACACTTTGACCCTTTATCATATCACACTTCCCATGTCTCCCTCTGCGAAGTGGGCACGGCGGGGGGCTGGAGCATTATGTAAACTGCACATGAAGTGTTTGGCGCAGTGCTTGGCATGGGATAAACACCAGTGAAGTAGCACTTAGGTGACACAGTGTTTCGCTGCATTTGTCACCAGTGCTATACCTTACTCATTTACTCATCTTCTTATTCCTGTCGCCTGGCACTGCATTGGAACAAAGAAATACACATATCTGTTTAAACTGAACTCTAGAAAGATTTGTGTCCAAAATAACAATATTTTATATTTTGATGCTGCAAAGCTGACACTTCTGGGTTTTTTTTTTTCCTTGCCAAGTTTCTTCTGCACCCAGCTCATTCTCCAGGGGCACATGGCAGTGGCTGGGCATAACTCTGGGTGTGCCGGCTCCCATGGTCTGCATTTCTAAGCAGTAGGGTGCAGTCAGCAAGGAGCCTGTGATGGGAGCCTGTGCCAGGGCAAGGCTGGGGCCATGCTGCTGCCTGCTGGCAGGAGTGGGGGTCCCAGCCTTGACAGCCCCTGAACTGAACGGGCCTTTCTGGCCATCCAGCTCATTCCAGGGTCCTGAGGCCACCTCTTCCTCTCGCCTCATTCTGCCTCTTGCACTTCTCTTGCAGAAATGTCCAATCTCGAGGATGGGGACGAGGGCAGAGGAAGTCTGCTAACATGCGGTGACGTCGAGGAGAATCCTGGCCCAGCACCGGGATCCATGGTGAGCAAGGGCGAGGAGCTGTTCACCGGGGTGGTGCCCATCCTGGTCGAGCTGGACGGCGACGTAAACGGCCACAAGTTCAGCGTGTCCGGCGAGGGCGAGGGCGATGCCACCTACGGCAAGCTGACCCTGAAGTTCATCTGCACCACCGGCAAGCTGCCCGTGCCCTGGCCCACCCTCGTGACCACCTTCACCTACGGCGTGCAGTGCTTCGCCCGCTACCCCGACCACATGAAGCAGCACGACTTCTTCAAGTCCGCCATGCCCGAAGGCTACGTCCAGGAGCGCACCATCTTCTTCAAGGACGACGGCAACTACAAGACCCGCGCCGAGGTGAAGTTCGAGGGCGACACCCTGGTGAACCGCATCGAGCTGAAGGGCATCGACTTCAAGGAGGACGGCAACATCCTGGGGCACAAGCTGGAGTACAACTACAACAGCCACAAGGTCTATATCACCGCCGACAAGCAGAAGAACGGCATCAAGGTGAACTTCAAGACCCGCCACAACATCGAGGACGGCAGCGTGCAGCTCGCCGACCACTACCAGCAGAACACCCCCATCGGCGACGGCCCCGTGCTGCTGCCCGACAACCACTACCTGAGCACCCAGTCCGCCCTGAGCAAAGACCCCAACGAGAAGCGCGATCACATGGTCCTGCTGGAGTTCGTGACCGCCGCCGGGATCACTCTCGGCATGGACGAGCTGTACAAGTGATAATCTAGAGGGCCCGTTTAAACCCGCTGATCAGCCTCGACTGTGCCTTCTAGTTGCCAGCCATCTGTTGTTTGCCCCTCCCCCGTGCCTTCCTTGACCCTGGAAGGTGCCACTCCCACTGTCCTTTCCTAATAAAATGAGGAAATTGCATCGCATTGTCTGAGTAGGTGTCATTCTATTCTGGGGGGTGGGGTGGGGCAGGACAGCAAGGGGGAGGATTGGGAAGACAATAGCAGGCATGCTGGGGATGCGGTGGGCTCTATGGATAACTTCGTATAATGTATGCTATACGAAGTTATACGCGTATAACTTCGTATAATGTATGCTATACGAAGTTATCAACGGGGTGAAACAGATAAAGACTCTCATAGAAAAAACAAACGAAGAGCGCAAGACACTGCTCAGCAACCTAGAAGAAGCCAAGAAGAAGAAAGAGGTCAGGAGGAGCCGCTACCGCCTCCCTGCCTTGACCATCCCACTGGAGGGGAGGGAGGGGGTCACTGCGCGGTGCCCTGCTGGGTTGCCATGGTGACCCGCAGTCCTCCCAGGCTGTGTCAGCTGATGCTGAGGCTGCAATTAAGAAGCAGGGAAGGTTCATTTGCTTCTGAAAGCATCAGGGAGTGAGATCTTGGATCTGGTTTTGTTATGAGCCTGGCCCAGGGCCTAATGCCCAGATTCATTTCAATAGATGTTTCTAAGCCCTGATCACATGCTAGTTCCAAGCAGGCTCTGGATGGGGTGGCGGCAGGGGCCCAGACAGGCGTGGCGTCCAACCTTCAGGAAGCTTATCTAATAGGGGTGATATGGTTAGGGTTAAGGCAGGCACACAGCACCCCACGTCTTGCTAGAGTTCTGGACTACTTCATAAAGAAACATCTTAAGCCCAGTAGTGGATATAGCAGGTCTAGCCTGTGCTTAATGATTCAACAAAACTAAAACACTATAATTCAATGAACAACTTATTTAAGAAATCAAGTCTCCTTAAGAGTATCCTACAAACCTCCTCTCACGGTCGCCCTACTTCAGATGCTAAGATGTAGCTACCTGCAAGCTTCATGATCCATCGTGCATGTTAAAAGCATTTCAAAG |

1. **Supplementary Figures**


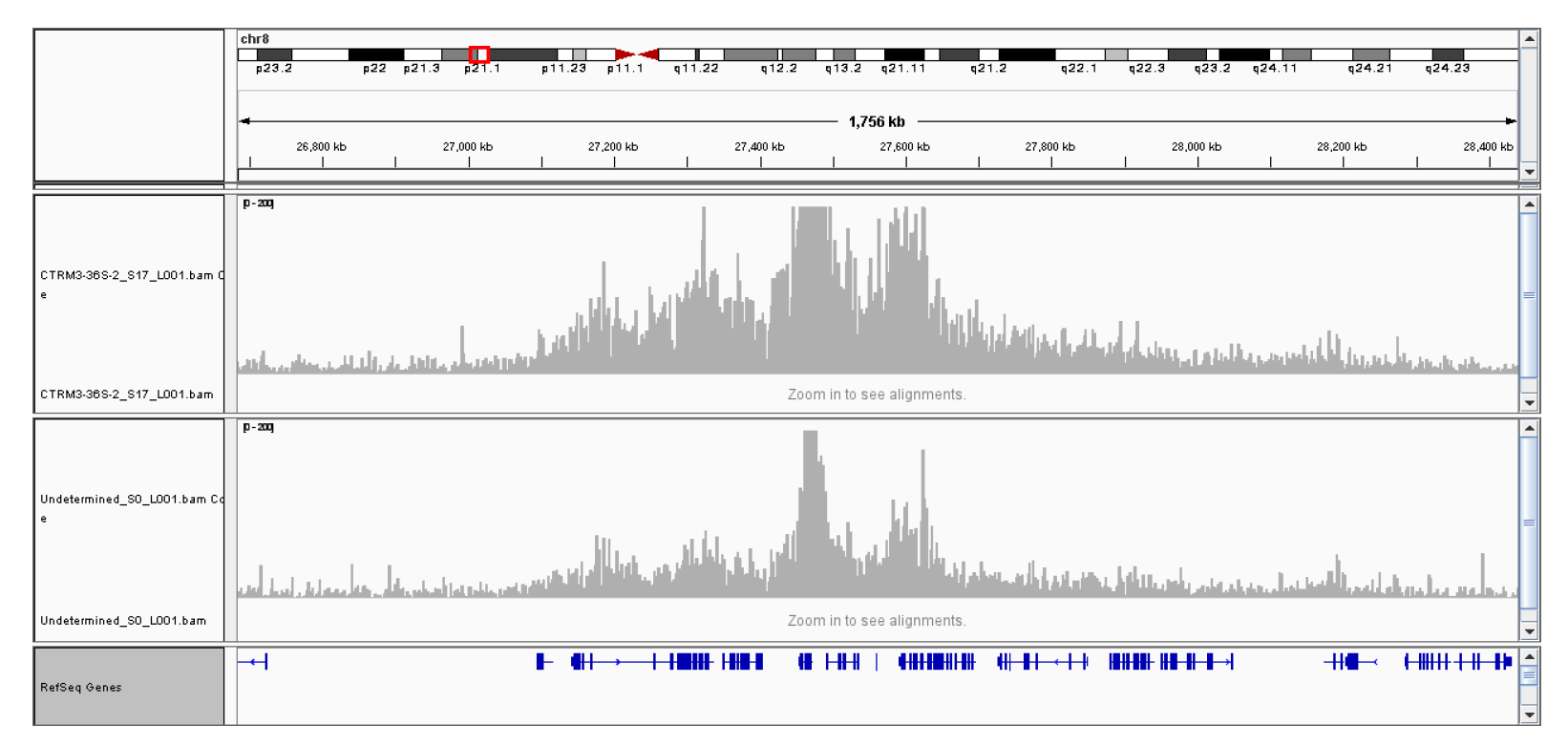


Supplementary Figure 1. No large deletions or other rearrangements were detected surrounding the integration site. Coverage profile surrounding transgene integration site. A coverage profile generated for the area surrounding the integration site on chromosome 8 showed no evidence for large deletions or any other rearrangements of chromosome 8.


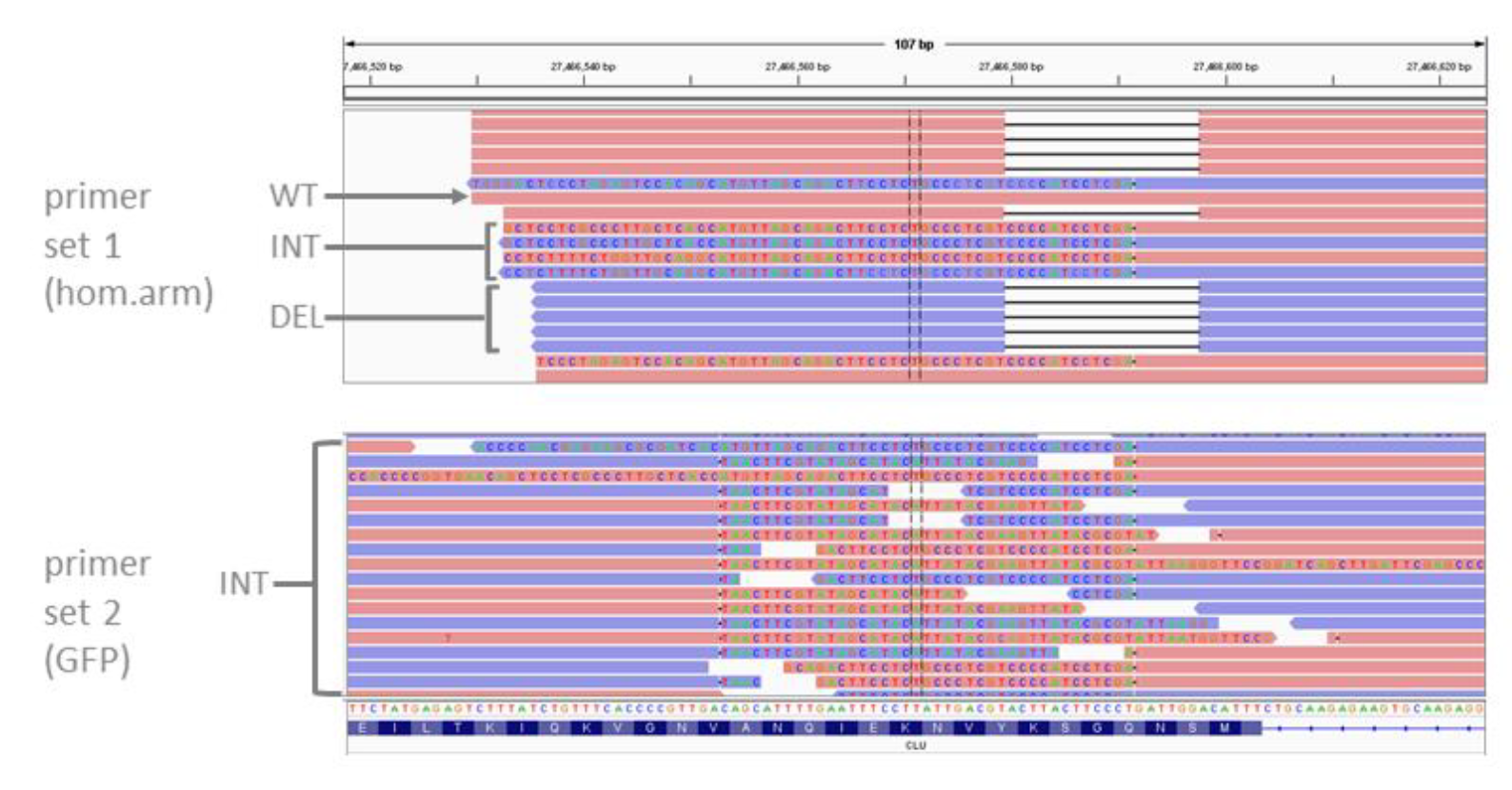


Supplementary Figure 2. Plot of sequence information generated on the position of the integration site shows reads generated with primer pair 1 and 2. WT = Wild type sequence, INT = expected integration, DEL = deletion. Primer set 1 reads inside one of the homology arms detecting INT, DEL and WT. Primer set 2 reads INT (Plot from Integrative Genomics Viewer). The sequence information generated on the position of the integration site (in between the two arms of homology) distinguished three types of reads at this site. Firstly there were reads from the integration (INT), which were expected to be found in the case of a clean integration. Secondly there were reads containing a deletion (DEL) spanning from position 27,466,579 to 27,466,589 (within the CRISPR site). One end of the deletion, position 27,466,579 lies outside of the arms of homology and is not present in the targeting construct. This demonstrates that the deletion is located in a different allele than the construct integration. This deletion produced a frame-shift in the CLU gene and introduced an early stop codon. Thirdly, primer set 1, which amplified both from the endogenous locus and from the targeting construct, found reads from the wild type allele (WT), fully aligning to the genome sequence at this position. The number of wild type reads was significantly lower than the insertion and deletion reads. The wild type constitutes ~ 1% of the reads. This low amount of wild type reads may come from a small number of wild type or heterozygous cells present in the sample. The cell line was recloned to eliminate potentially contaminating wild type cells.


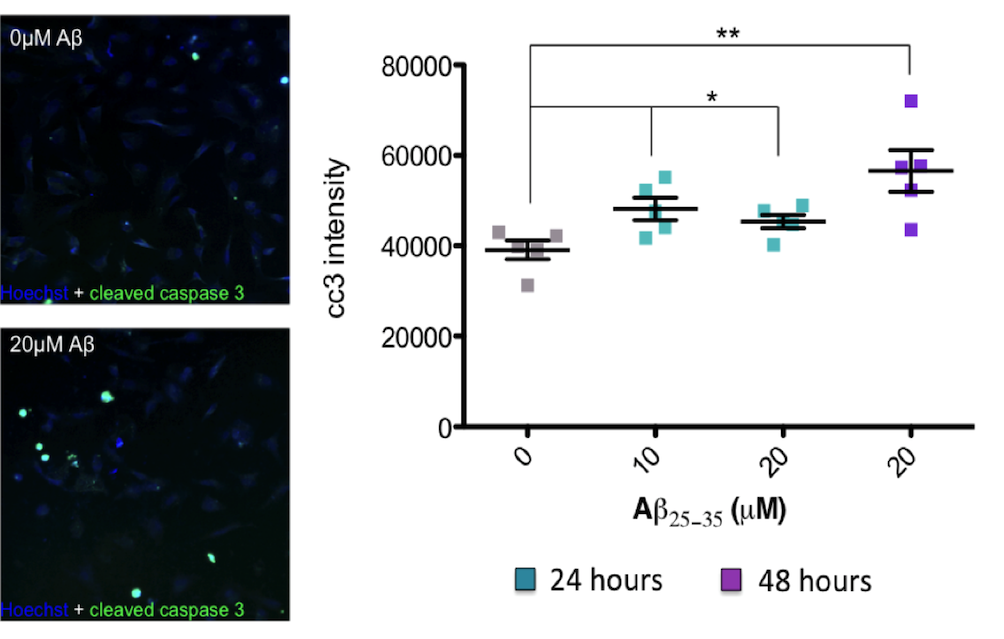


Supplementary Figure 3. Aβ_25-35_ treatment increases apoptosis at 24 hours. Neurons differentiated in 5 individual plates from a single differentiation experiment were treated with Aβ_25-35_ or H_2_0 control for 24 or 48 hours. Cells were stained with cleaved caspase 3 (cc3) antibody (1:500) and Hoechst and plates were imaged with cc3 staining intensity across each well measured. Image panels show neurons stained with Hoechst and cc3, untreated and treated with 20μM for 48 hours. (*=p<0.05, **=p<0.005)


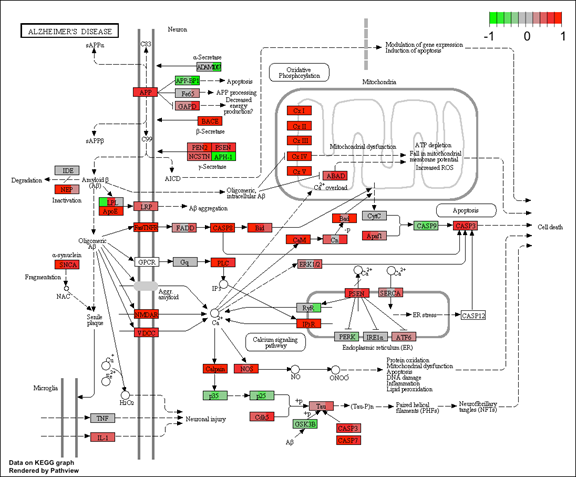


Supplementary Figure 4. The Alzheimer’s disease pathway is significantly upregulated in CLU-untreated compared to WT-untreated cells. The scale bar shows upregulated genes (closer to 1) are labelled in red and downregulated genes (closer to -1) are labelled in green. Interestingly, this disease pathway is downregulated in CLU-Aβ neurons compared to CLU-untreated neurons, but not during Aβ-treatment in wildtype neurons, which may have a role in the neuroprotection observed in the CLU-Aβ neurons.

**
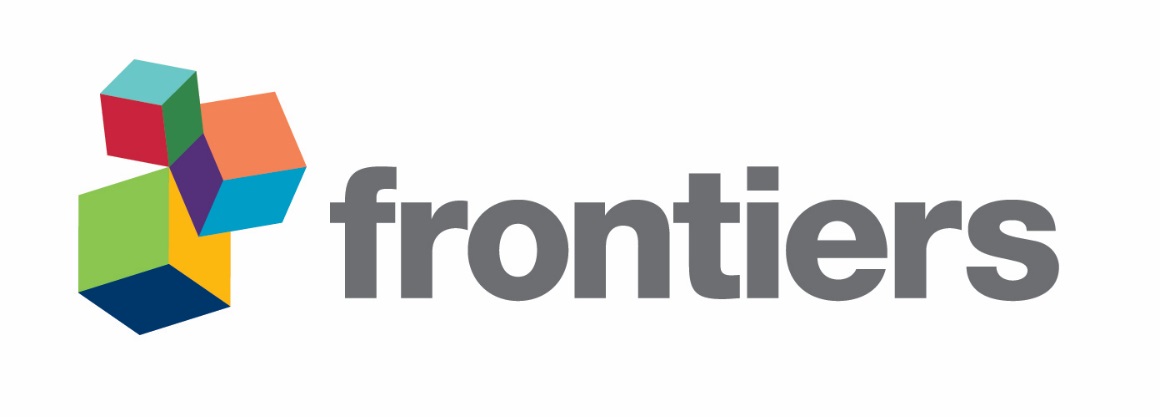
**
